# Species-specific IgE recognition of cockroach allergens in human–dog dyads sharing the same household

**DOI:** 10.3389/falgy.2026.1932833

**Published:** 2026-10-07

**Authors:** Mey-Fann Lee, Jane Lu Hsu, Ching-Chun Lin, Yu-Wen Chu, Nancy M. Wang

**Affiliations:** 1 Department of Medical Research, Taichung Veterans General Hospital, Taichung, Taiwan; 2 Department of Marketing, National Chung Hsing University, Taichung, Taiwan; 3 Department of Chemistry, Tunghai University, Taichung, Taiwan; 4 Department of Pharmacy, College of Pharmaceutical Sciences, National Yang Ming Chiao Tung University, Taipei, Taiwan; 5 Department of Pharmacy, Taichung Veterans General Hospital, Taichung, Taiwan; 6 Department of Biology, National Changhua University of Education, Changhua, Taiwan

**Keywords:** cockroach allergens, human-dog dyads, immunoblot, One Health, species-specific IgE

## Abstract

**Introduction:**

Allergic diseases commonly affect both humans and companion animals, yet little is known about how shared household environments influence allergen recognition across species. Cockroach allergens are major contributors to human allergic disease, yet their immunological impact on cohabiting dogs has not been systematically evaluated. Using a One Health–informed comparative approach, we investigated cockroach IgE recognition profiles in 68 human–dog dyads residing in shared domestic environments.

**Methods:**

We assessed household characteristics, allergic histories, and cockroach infestation patterns through structured questionnaires. Serum IgE reactivity to *Periplaneta americana* allergens was analyzed by immunoblotting in paired owner–dog samples.

**Results:**

Concurrent allergic conditions were identified in 52.9% of owner–dog pairs. Cockroach activity was most frequently reported in kitchens (61.4%), with additional evidence near dog feeding bowls (10.0%) and sleeping areas (4.3%), indicating common environmental exposure. Comparative immunoblot analysis revealed both shared and species-specific IgE recognition patterns. Per a 1, Per a 3, Per a 4, and Per a 9 were recognized in both species, whereas dogs showed higher recognition frequencies for Per a 4 (69%) and Per a 9 (40%), while owners more frequently recognized Per a 1 (34%), Per a 3 (34%) and Per a 7 (26%).

**Discussion:**

These findings suggest that shared household exposure does not necessarily produce identical allergen recognition profiles and support the concept of species-specific allergen recognition under common environmental conditions. This study provides new insights into comparative allergy between humans and companion dogs and highlights the importance of considering both host biology and household exposure when investigating environmental allergic diseases.

## 1 Introduction

The domestic environment has become a shared micro-ecosystem in which humans and companion animals are simultaneously exposed to common environmental factors. This concept aligns with the One Health framework, which recognizes that the health of humans, animals, and their shared environment is closely interconnected (1–4). As pet ownership continues to increase worldwide, there is growing interest in understanding how shared indoor environments contribute to the development of allergic diseases across species (3, 5–7). Identifying common environmental risk factors may improve our understanding of allergy pathogenesis while informing strategies for disease prevention in both humans and companion animals.

Among indoor environmental allergens, cockroach-derived allergens, particularly those from *Periplaneta americana* (the American cockroach), are well-established triggers of allergic rhinitis, asthma, and atopic dermatitis in humans (8–12). Advances in component-resolved diagnostics have identified ten major allergenic molecules (Per a 1–Per a 10), enabling detailed characterization of allergen-specific IgE responses and improving the precision of allergy diagnosis (8, 10, 13). Although the role of cockroach allergens in human allergic disease has been extensively investigated, considerably less is known about their contribution to allergic sensitization in companion animals exposed to the same indoor environment.

Domestic dogs frequently develop naturally occurring allergic diseases, particularly canine atopic dermatitis, which shares many clinical, immunological, and pathological characteristics with human atopic disease, including IgE-mediated sensitization to environmental allergens (14, 15). Because dogs inhabit the same indoor environment as their owners while exhibiting distinct behavioral patterns, such as increased contact with floor surfaces, household dust, and organic debris around feeding and resting areas, they may experience different allergen exposure profiles despite living in the same household. Consequently, dogs have been proposed as biological sentinels of indoor environmental quality and represent a valuable comparative model for investigating environmental allergen exposure within a One Health framework (6, 16). However, whether shared household exposure results in similar or species-specific recognition of individual cockroach allergen components remains unknown. To date, no study has systematically compared molecular IgE recognition profiles to *P. americana* allergens between cohabiting humans and their pet dogs.

To address this knowledge gap, we conducted a One Health study involving 68 owner–dog pairs living in the same household. Household cockroach exposure was assessed using a structured environmental questionnaire, and serum IgE reactivity against *P. americana* allergens was analyzed by immunoblotting. We aimed to determine the frequency of cockroach allergen-specific IgE recognition, compare molecular recognition profiles between humans and dogs, and evaluate the relationship between household environmental exposures and allergen-specific IgE responses. This study provides novel insights into cross-species allergen recognition patterns and highlights the value of integrating human and veterinary perspectives to better understand the environmental determinants of allergic diseases.

## 2 Materials and methods

### 2.1 Ethical approval

The study protocol was approved by the Institutional Review Board of Taichung Veterans General Hospital (IRB TCVGH No: CE23070A-1/-2), and all aspects of the study conformed to the ethical guidelines of the 1975 Declaration of Helsinki. All human participants provided written informed consent before enrollment. Ethical approval was not required for pet dogs, as licensed veterinarians collected blood during routine clinical procedures.

### 2.2 Study participants and recruitment

Participants were recruited between September 2024 and July 2025 from the outpatient lobby of Taichung Veterans General Hospital, Taiwan. Dog owners visiting the hospital for medical services were approached consecutively and invited to participate if they owned at least one pet dog living in the same household. Recruitment was conducted by trained research personnel using face-to-face screening. Eligible participants who agreed to participate completed the questionnaire and provided blood samples. Their pet dogs subsequently underwent blood collection at a collaborating veterinary clinic. Each enrolled owner–dog pair represented a shared household environment for subsequent analyses.

### 2.3 Study design and questionnaire-based environmental assessment

A total of 68 human–dog dyads residing in shared household environments were enrolled to investigate patterns of allergen-specific IgE responses. To characterize the household environment and exposure levels, owners completed a structured questionnaire designed to capture demographic characteristics, allergic history, environmental exposures, and pet-related factors relevant to household allergen distribution. The survey assessed: (1) demographic characteristics of both species; (2) clinical history of allergies (e.g., dermatitis, respiratory symptoms); and (3) observed cockroach activity within the home. Specific attention was paid to “environmental interfaces” where dogs and cockroaches are likely to interact, such as kitchen areas, feeding bowls, and pet sleeping quarters.

### 2.4 Preparation of American cockroach extracts and *Escherichia coli*-expressed recombinant proteins

Crude extracts of *Periplaneta americana* (CraA) were prepared as previously described (8). These extracts served as a representative environmental allergen source reflecting household cockroach exposure.

Recombinant Per a 1-10 proteins, except Per a 8, were produced in our laboratory using *Escherichia coli* expression systems. Following bacterial expression, the recombinant proteins were purified by affinity chromatography using reagents supplied by Novagen (Darmstadt, Germany), according to our previously established protocols (8, 10). Per a 8 was excluded because it was infrequently detected in CraA; therefore, nine recombinant Per a allergens were included in the present analysis.

### 2.5 IgE immunoblotting and component-resolved analysis

Allergen-specific IgE reactivity to cockroach extracts was assessed by immunoblotting to enable comparative analysis across species. CraA proteins were separated by SDS–PAGE (4% stacking gel, 12% resolving gel) and transferred onto nitrocellulose membranes. Membranes were blocked with 2% bovine serum albumin in PBST and incubated overnight at 4 °C with individual sera from owners or dogs (1:5 dilution). Species-specific detection was performed using mouse anti-human IgE alkaline phosphatase–conjugated antibodies or mouse anti-canine IgE horseradish peroxidase–conjugated antibodies. Following washing, immunoreactive bands were visualized using enhanced chemiluminescence (Millipore, MA, USA) and recorded with a high-resolution gel imaging system (G:Box Chemi-XX9, SYNGENE).

Previously characterized polyclonal antibodies against nine selected Per a allergens (Per a 1–7 and Per a 9–10) were used as reference profiles to assist in identifying corresponding allergen bands in CraA (8). Per a 8 was excluded because of its low abundance and infrequent detection in CraA; therefore, nine allergen-specific antibodies were used. For each antibody, two lanes were analyzed: the left lane contained the corresponding purified recombinant Per a allergen as a positive reference, whereas the right lane contained CraA, allowing identification of the migration position of the corresponding native allergen component. These reference profiles were subsequently used to assist in assigning putative Per a allergen bands in the serum IgE immunoblots.

Because the volume of serum that could be ethically collected from pet dogs was limited, the available canine samples were prioritized for qualitative immunoblot analyses. Comprehensive immunoblot inhibition assays, quantitative canine allergen-specific IgE measurements, and validation of all allergen components were therefore not feasible. Accordingly, the present study focused on comparative IgE recognition patterns between paired human and dog sera rather than on quantitative assessment of canine allergen-specific IgE responses. Accordingly, the present study focused on comparative IgE recognition rather than comprehensive characterization of the canine allergen repertoire.

### 2.6 Statistical analysis

Statistical analyses were performed using SPSS Statistics version 22. Allergy prevalence was expressed as percentages. Chi-squared tests were used to assess associations between allergic status in owners and their dogs, reflecting shared household exposure. Logistic regression analysis was applied to evaluate the relationship between environmental factors and allergy prevalence across species. A *p-*value of < 0.05 was considered statistically significant.

## 3 Results

### 3.1 Household-based characteristics of human–dog dyads

A total of 68 owner–dog pairs sharing the same household environment were included in this study. The demographic and allergy-related characteristics of owners and their pets are summarized in Table 1. Among owners, the mean age was 34.10 ± 10.84 years, with a predominance of female participants (79.41%). A history of allergic disease was reported by 82.35% of owners. Notably, 16.18% of owners self-reported a worsening of allergic symptoms following the introduction of a pet dog into the household, suggesting a perceived interaction between pet ownership and allergic disease expression in a shared indoor environment. Among pet dogs, 66.18% were reported by their owners to have experienced allergic symptoms.

**Table 1:** Demographic and allergy characteristics of dog owners and their pets (*N* = 68).

Characteristic	Owners (*n* = 68)	Dogs (*n* = 68)
Mean age, years (±SD)	34.10 (±10.84)	5.54 (±3.81)
Sex, %, male/female	20.59/79.41	45.59/54.41
Residential duration, years (±SD)	8.83 (±10.64)	–
History of allergy, %	82.35	66.18
Self-reported worsening of allergy after dog ownership, %	16.18	–

When allergy status was examined at the household level, more than half of the dyads (52.94%, 36/68) exhibited concurrent allergic conditions in both the owner and the dog (Table 2). This high rate of co-occurrence suggests that shared household environmental exposure may contribute to the allergic health of the multi-species household. Dyads in which only the owner reported allergies accounted for 29.41%, while dyads with allergic dogs but non-allergic owners comprised 13.23%. Only 4.41% of households reported no allergic symptoms in either species. These findings indicate a high degree of overlap in allergic manifestations within shared domestic environments.

**Table 2:** Distribution of sample numbers in the owner–dog allergy matrix by owner self-assessment (*N* = 68).

Allergic disease status in dogs	Owner without self-reported allergy	Owner with self-reported allergy
Dog without allergic disease	*N* = 3 (4.41%)	*N* = 20 (29.41%)
Dog with allergic disease	*N* = 9 (13.23%)	*N* = 36 (52.94%)

### 3.2 Environmental evidence of household cockroach exposure

Questionnaire-based assessment revealed widespread household exposure to cockroaches among participating human–dog dyads (Table 3). Cockroach activity was most frequently reported in kitchens (61.43%), followed by living rooms (30.43%), bedrooms (18.84%), and bathrooms (11.59%). In addition to general household areas, evidence of cockroach activity was reported in dog-associated micro-environments, including areas near feeding bowls (10.00%) and dog sleeping areas (4.29%), indicating potential shared exposure pathways for both humans and companion animals within the same domestic setting.

**Table 3:** Household cockroach exposure characteristics in human–dog shared environments (*N* = 68).

Characteristic	*n* (%)
Section A. Household pest management and dog-related factors	
Cockroach bait placed in household	42.86
Dog observed catching cockroaches	31.88
Increased cockroach sightings after dog ownership	8.57
Section B. Locations of cockroach infestation within households	
Kitchen	61.43
Living room	30.43
Bedroom	18.84
Bathroom	11.59
Section C. Evidence of cockroach activity in dog-associated areas	
Near dog feeding bowls	10.00
Near dog sleeping areas	4.29

Data are based on owner-reported questionnaire responses. Percentages are calculated using the total number of households (*N* = 68). Multiple responses were allowed.

Regarding household pest management and animal-related factors, 42.86% of owners reported placing cockroach bait in the household, while 31.88% observed their dogs catching cockroaches. Among the owners, 8.57% reported more frequent cockroach sightings after dog ownership. This observation may reflect changes in household environmental conditions associated with pet keeping, including the availability of pet food residues and other organic materials around feeding areas. When feeding areas and food residues are not adequately cleaned, such conditions may provide additional food resources for cockroaches and potentially increase their activity or visibility. However, because cockroach infestation was not objectively quantified in the present study, this association should not be interpreted as evidence that dog ownership directly causes or increases cockroach infestation.

Taken together, the questionnaire data indicate that allergic symptoms in humans and dogs frequently co-occur within the same household and are associated with shared environmental exposure to cockroach infestation.

### 3.3 Comparative IgE recognition profiles to cockroach allergens

To characterize molecular IgE recognition patterns, allergen-specific IgE reactivity to *Periplaneta americana* crude extracts (CraA) was examined in sera from owners and their pet dogs. Representative immunoblotting profiles from 56 owner–dog pairs are shown in Supplementary Figure S1. As shown in Figure 1A, polyclonal antibodies against Per a 1–10, except Per a 8, were first used to identify the corresponding allergen positions in the CraA. Based on these reference positions, serum samples from owners and their dogs were subsequently analyzed to establish their IgE-binding profiles, with representative results shown in Figures 1B,C, respectively.

**Figure 1:**
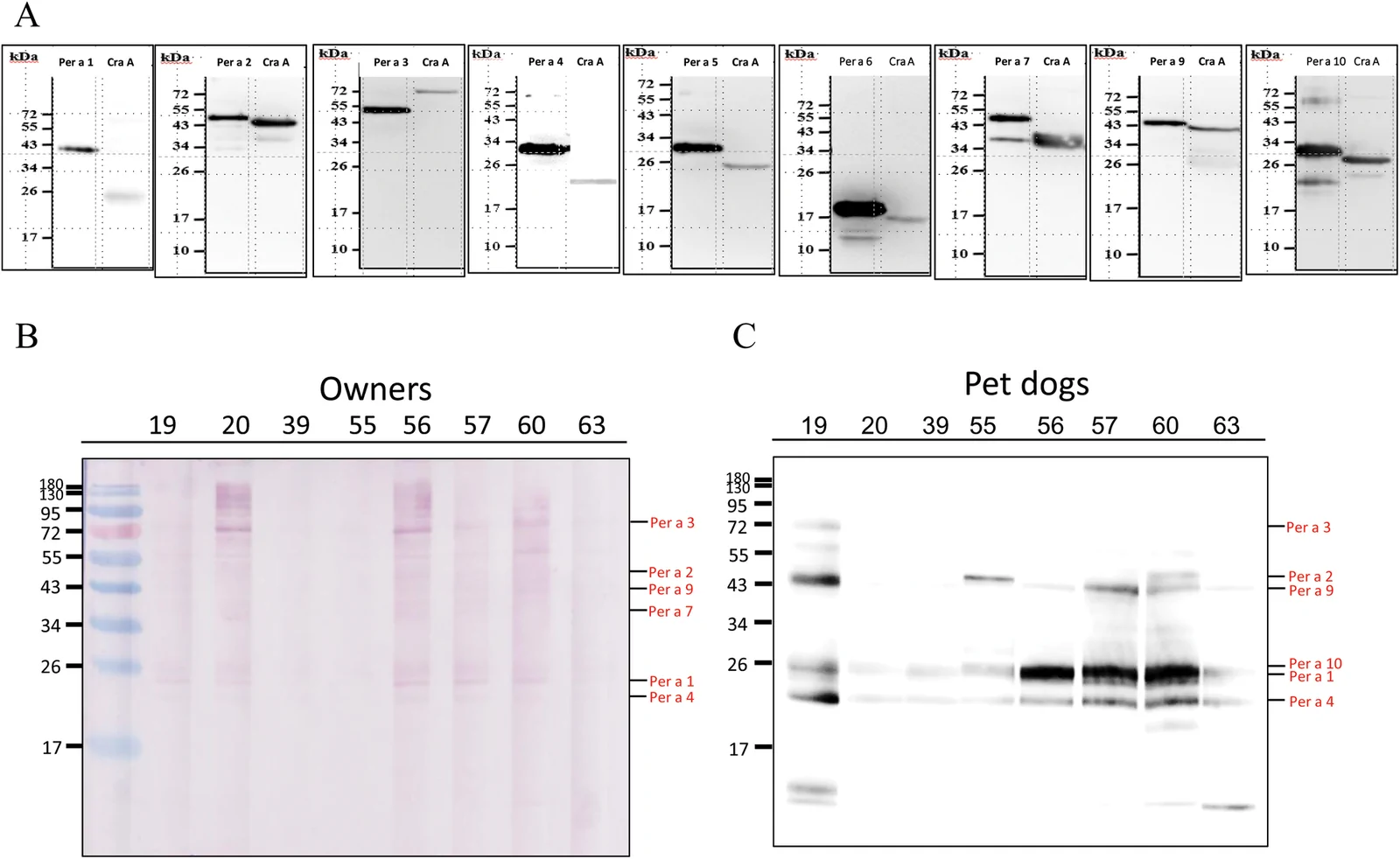
Comparative IgE recognition of *Periplaneta Americana* crude allergen extract (CraA) in human owners and their pet dogs. **(A)** Reference immunoblots obtained using individual rabbit antisera against selected Per a allergens (Per a 1–Per a 10, except Per a 8). For each antiserum, the left lane shows reactivity to the corresponding purified recombinant Per a allergen as a positive reference, whereas the right lane shows reactivity to CraA, indicating the migration position of the corresponding native allergen component. Per a 8 was excluded because of its low abundance and infrequent detection in CraA. These reference profiles were used to assist in identifying putative Per a allergen components in subsequent IgE immunoblots. **(B)** Representative IgE-binding profiles of sera from human owners against CraA. **(C)** Representative IgE-binding profiles of sera from their cohabiting pet dogs against CraA. The positions of the selected Per a allergens are indicated based on the reference immunoblots shown in panel A.

Immunoblot analysis identified several major IgE-reactive cockroach allergen components that were recognized by paired human and canine sera. The frequencies of IgE recognition of individual cockroach allergen components are summarized in Figure 2. Among owners, the most frequently recognized allergens were Per a 1 (34%) and Per a 3 (34%), followed by Per a 7 (26%) and Per a 4 (18%). IgE reactivity was also detected against Per a 2 (9%) and Per a 9 (9%), whereas no detectable IgE reactivity was observed against Per a 5, Per a 6, or Per a 10. In contrast, pet dogs exhibited markedly higher IgE recognition frequencies for Per a 4 (69%), Per a 1 (63%), and Per a 9 (40%). Reactivity to Per a 3 (9%) and Per a 10 (6%) was comparatively low in dogs. Minimal or no IgE binding to Per a 5 and Per a 6 was observed in either species.

**Figure 2:**
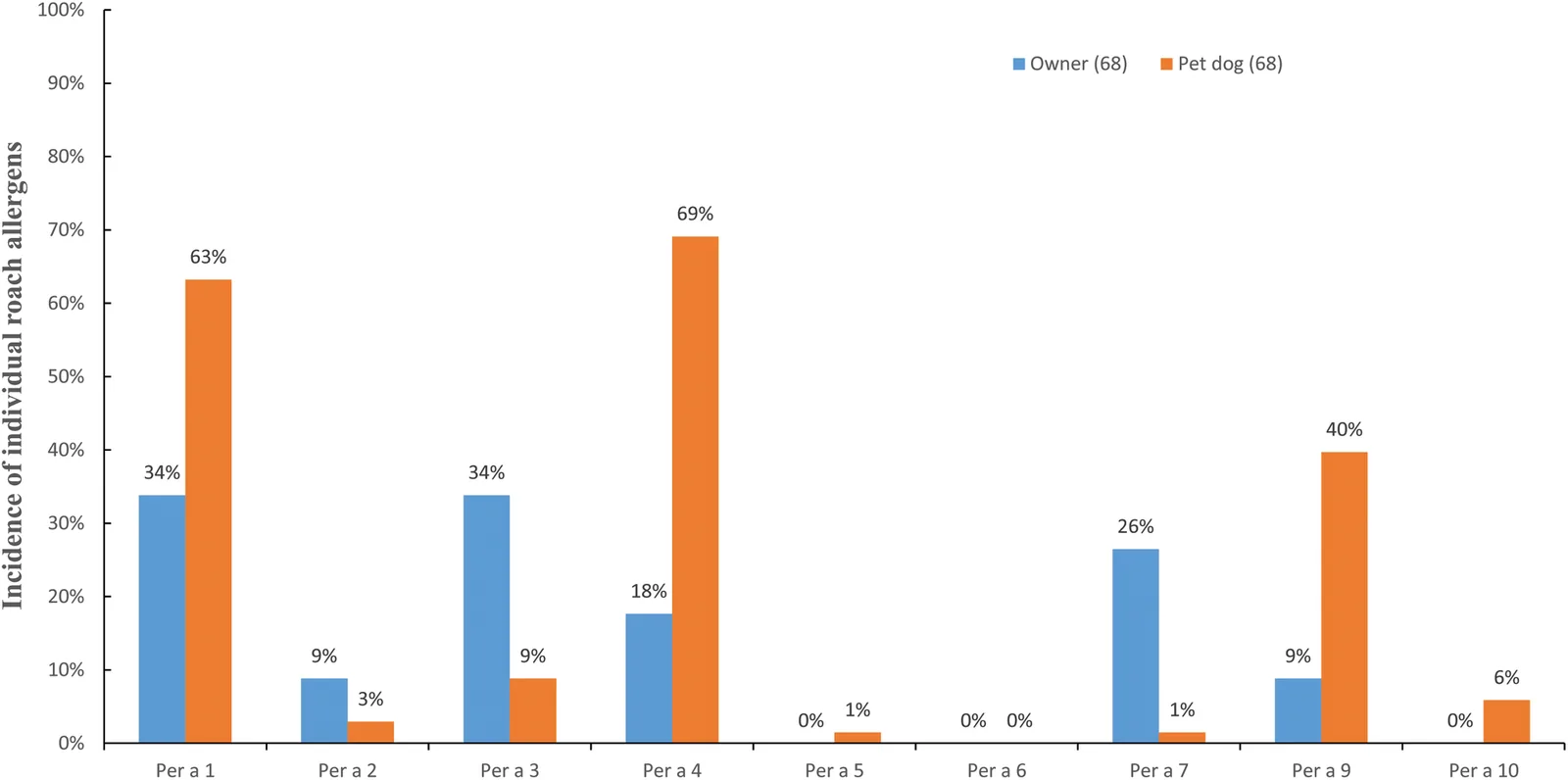
Prevalence of IgE recognition of selected *P. Americana* allergen components. The bar chart illustrates the percentage of owners (blue) and pet dogs (orange) showing IgE recognition of CraA. The data demonstrate both overlapping and species-specific IgE recognition patterns, with higher recognition frequencies for Per a 4, Per a 1, and Per a 9 in dogs, whereas owners showed higher IgE recognition frequencies for Per a 1, Per a 3, and Per a 7. Per a 1 was also frequently recognized in both species, with a higher recognition frequency in dogs than in owners. Percentages were calculated based on the total cohort of 68 human-dog pairs.

Taken together, these findings indicate that the major overlapping IgE-recognized allergens between owners and dogs are Per a 1, Per a 3, Per a 4, and Per a 9**.** Notably, dogs showed higher IgE recognition frequencies for Per a 4, Per a 1, and Per a 9**,** whereas owners showed higher IgE reactivity recognition frequencies for Per a 3, and Per a 7, highlighting both shared and distinct IgE recognition patterns between the two species.

## 4 Discussion

This study provides a comparative analysis of cockroach allergen recognition in cohabiting human–dog pairs. By integrating comparative IgE immunoblot analysis with household environmental survey data, we demonstrated that although owners and their pet dogs frequently shared allergic conditions, they exhibited distinct allergen recognition profiles. To our knowledge, this is the first study to characterize species-specific IgE recognition patterns to American cockroach allergens in paired humans and companion dogs living in the same household.

### 4.1 The shared household as a one health micro-ecosystem

Our findings underscore the concept of the household as a shared micro-ecosystem where humans and animals are subject to the same environmental stressors. The high rate of concurrent allergic conditions (52.92%) within the dyads suggests that cockroach infestation is not merely a human health issue but a pervasive environmental hazard affecting all mammalian inhabitants. The kitchen was identified as the primary reservoir for *P. americana* (61.43%), but the identification of cockroach activity near feeding bowls (10%) and sleeping areas (4.29%) reveals a specific exposure pathway for dogs. Our results are consistent with previous studies showing a positive correlation between domestic cockroach exposure and the likelihood of cockroach allergen-specific IgE recognition (5, 17). These behaviors may increase dogs' opportunities for repeated contact with household dust and surfaces potentially contaminated with cockroach allergens.

Because environmental allergen concentrations could not be reliably quantified, household cockroach exposure in the present study was evaluated using standardized questionnaires describing infestation sites and owner-reported evidence of cockroach activity. These measures represent surrogate indicators of environmental exposure rather than direct allergen quantification.

### 4.2 Species-specific molecular signatures: per a 4 and per a 9 in dogs

A pivotal discovery of this research is the interspecies discrepancy in IgE recognition profiles toward allergens Per a 1–10. While Per a 1 and Per a 3 were common antigens for both species, pet dogs exhibited a strikingly higher prevalence of IgE reactivity to Per a 4 (69%) and Per a 9 (40%). In contrast, owners showed higher IgE recognition frequencies for Per a 3 and Per a 7. These differences likely result from multiple biological processes, including species-specific exposure behaviors, epithelial barrier function, antigen uptake, and immune processing (18, 19).

The predominance of Per a 4 (a lipocalin-like protein) and Per a 9 (arginine kinase) recognition in dogs may reflect differences in exposure behavior rather than differences in household allergen sources. Dogs spend prolonged periods in close contact with floor surfaces through sniffing, licking, resting, and feeding behaviors, increasing their opportunity for repeated exposure to household dust and cockroach-associated microenvironments (20–22). In support of this observation, dogs from households reporting cockroach activity near feeding bowls showed higher frequencies of IgE recognition to Per a 4 and Per a 9. Although environmental allergen concentrations were not directly measured in the present study, these findings suggest that shared household exposure does not necessarily result in identical allergen recognition profiles. Instead, species-specific behavioral exposure, together with differences in antigen processing and immune responses, may contribute to the distinct IgE recognition patterns observed between humans and dogs (23, 24).

### 4.3 Potential differences in allergen exposure and IgE recognition pathways between humans and dogs

Although humans and dogs shared the same household environment, their allergen-specific IgE recognition profiles differed considerably, suggesting that environmental exposure alone does not fully explain species-specific IgE responses observed between the two species. Instead, differences in allergen exposure routes, host biological characteristics, and behavioral patterns may contribute to differential allergen recognition.

One possible explanation is the difference in the primary route of allergen exposure. In humans, inhalation is generally regarded as an important route of exposure to indoor aeroallergens, including cockroach allergens, with effects on the upper and lower respiratory tracts. In contrast, companion dogs frequently contact floor surfaces through walking, lying, sniffing, and grooming, resulting in frequent contact of the skin and oral mucosa with environmental materials that may contain allergens. Experimental studies have demonstrated that epicutaneous allergen exposure can induce allergen-specific IgE responses and promote allergic inflammation, particularly when epithelial barrier integrity is impaired (9, 12).

Species-specific characteristics of the epithelial barrier may further influence allergen uptake and subsequent IgE recognition. Canine atopic dermatitis is associated with impaired skin barrier function, altered lipid composition, and increased transepidermal water loss, changes that may facilitate allergen penetration and immune activation (25). Similar mechanisms have been described in human atopic dermatitis, although the relative contribution of respiratory and cutaneous exposure may differ between species (15). Consequently, exposure to the same household allergen source may result in differences in antigen uptake, processing, and IgE recognition.

In addition, differences in innate immune responses, antigen presentation, and behavioral exposure may further shape allergen recognition profiles. These factors may contribute to the higher frequencies of IgE recognition of Per a 4 and Per a 9 observed in dogs, whereas owners showed greater IgE recognition of Per a 3 and Per a 7. However, because the present study did not directly assess allergen exposure routes, epithelial barrier function, antigen presentation, or IgE production, these mechanisms should be considered hypotheses rather than experimentally demonstrated pathways. Future studies integrating standardized environmental allergen quantification, epithelial barrier biomarkers, and longitudinal monitoring of human–dog dyads will be valuable for clarifying the biological mechanisms underlying the distinct IgE recognition patterns observed between species.

### 4.4 Dogs as biological sentinels of household allergen exposure

From a comparative allergy perspective, our findings suggest that pet dogs may serve as biological sentinels of household allergen exposure. Dogs spend prolonged periods in close contact with floor surfaces, household dust, and pet-associated microenvironments, resulting in greater cumulative exposure to environmental allergens than their owners. Consequently, alterations in canine IgE recognition profiles may reflect changes in household allergen burden before similar patterns become clinically apparent in humans (12, 26). Although additional longitudinal studies are required, our findings support the hypothesis that companion dogs may function as practical biological sentinels for monitoring household allergen exposure and may complement conventional environmental assessments.

### 4.5 Implications for integrated pest and allergen management

The results of this study may have implications for public health and veterinary practice. Standard pest control measures often focus on visible human-centric areas. However, our study highlights the need for “Integrated Pest Management” that specifically addresses pet-related interfaces, such as regular cleaning of feeding stations and sleeping quarters. Reducing cockroach infestation in these hotspots is essential not only for managing canine atopic dermatitis but also for mitigating the cumulative allergen load within the shared domestic environment, thereby protecting the health of the entire dyad.

### 4.6 Limitations and future directions

A limitation relates to the restricted volume of canine serum that could be collected under ethical and clinical considerations. Consequently, analyses were prioritized for comparative immunoblotting, and comprehensive canine allergen-specific IgE quantification, immunoblot inhibition assays, and evaluation of additional allergen components were not feasible. Therefore, the present study characterizes comparative IgE recognition patterns rather than providing direct evidence of allergen cross-reactivity or the complete spectrum of canine cockroach allergen-specific IgE recognition.

A further limitation is that environmental cockroach allergen concentrations were not included in the final analysis. Household dust samples were collected by dog owners rather than by trained study personnel using a standardized sampling protocol. Consequently, substantial variability in sampling location, surface area, collection method, and the amount and quality of dust obtained limited the comparability and reliability of quantitative ELISA measurements. After evaluation of the available specimens and assay performance, these measurements were not considered sufficiently reliable for inclusion in the analysis. Therefore, the household questionnaire data should be interpreted as an assessment of reported cockroach exposure rather than a quantitative measure of environmental allergen burden. Future studies should employ standardized, prospectively supervised dust collection and validated quantitative allergen assays to directly relate environmental allergen levels to component-specific IgE recognition in human–dog dyads.

Although analysis of nine selected allergens could not fully capture the molecular IgE recognition profile of *P. americana*, for which 23 allergens are currently registered in the WHO/IUIS Allergen Nomenclature Database (http://www.allergen.org), it was sufficient to address the primary comparative objective of this study. Specifically, our findings demonstrate that humans and dogs sharing the same household environment exhibit distinct allergen-specific IgE recognition patterns.

Together, these findings suggest that paired human–dog investigations provide a useful translational model for understanding environmental allergen-specific IgE recognition and may facilitate future comparative studies in both veterinary and human allergy research. Such studies may help distinguish differences in exposure routes from intrinsic species-specific immune responses.

### 4.7 Conclusions

In conclusion, humans and their cohabiting pet dogs exhibit both shared and species-specific IgE recognition of American cockroach allergens despite living in the same household environment. These findings provide new insights into comparative allergy and support the use of paired human–dog studies to investigate household allergen exposure and allergic disease.

## Data Availability

The original contributions presented in the study are included in the article/Supplementary Material, further inquiries can be directed to the corresponding author.
