# Supplementary material for: Species-specific IgE recognition of cockroach allergens in human–dog dyads sharing the same household
Source: Front Allergy. 2026 Oct 7;7:1932833. doi: 10.3389/falgy.2026.1932833 (PMC13683157; doi:10.3389/falgy.2026.1932833)
Supplement: Supplementary file 1 [file Table1.docx]

**Supplementary Materials**

**Figure S1. Comparative IgE recognition patterns of cockroach allergen extract in paired human and dog sera.** Representative immunoblot profiles of *Periplaneta americana* crude allergen extract (CraA) obtained using sera from human owners (A) and their cohabiting pet dogs (B) are shown. Specific IgE recognition of CraA was evaluated in 68 paired human–dog sera. Proteins from CraA were separated by SDS–PAGE and transferred to nitrocellulose membranes, followed by incubation with individual serum samples. Previously characterized polyclonal antibodies against selected Per a allergens were used as reference profiles to assist in the identification of putative allergen bands. The immunoblot results demonstrate comparative IgE recognition patterns between cohabiting human–dog pairs, including both shared and species-specific allergen recognition, rather than providing comprehensive validation of all allergen components.
